# Knowledge Translation in Men’s Health Research: Development and Delivery of Content for Use Online

**DOI:** 10.2196/jmir.3881

**Published:** 2015-01-29

**Authors:** Maria Lohan, Áine Aventin, John L Oliffe, Christina S Han, Joan L Bottorff

**Affiliations:** ^1^School of Nursing and MidwiferyQueen's University BelfastBelfastUnited Kingdom; ^2^School of NursingDistinguished Scholar in Residence, Peter Wall Institute for Advanced StudiesUniversity of British ColumbiaVancouver, BCCanada; ^3^Honorary Professor, School of NursingUniversity of QueenslandBrisbaneAustralia; ^4^School of NursingUniversity of British ColumbiaVancouver, BCCanada; ^5^School of NursingUniversity of British ColumbiaKelowna, BCCanada; ^6^Professorial FellowFaculty of Health ScienceAustralian Catholic UniversitySydneyAustralia

**Keywords:** men’s health, knowledge translation, development of online content

## Abstract

**Background:**

Men can be hard to reach with face-to-face health-related information, while increasingly, research shows that they are seeking health information from online sources. Recognizing this trend, there is merit in developing innovative online knowledge translation (KT) strategies capable of translating research on men’s health into engaging health promotion materials. While the concept of KT has become a new mantra for researchers wishing to bridge the gap between research evidence and improved health outcomes, little is written about the process, necessary skills, and best practices by which researchers can develop online knowledge translation.

**Objective:**

Our aim was to illustrate some of the processes and challenges involved in, and potential value of, developing research knowledge online to promote men’s health.

**Methods:**

We present experiences of KT across two case studies of men’s health. First, we describe a study that uses interactive Web apps to translate knowledge relating to Canadian men’s depression. Through a range of mechanisms, study findings were repackaged with the explicit aim of raising awareness and reducing the stigma associated with men’s depression and/or help-seeking. Second, we describe an educational resource for teenage men about unintended pregnancy, developed for delivery in the formal Relationship and Sexuality Education school curricula of Ireland, Northern Ireland (United Kingdom), and South Australia. The intervention is based around a Web-based interactive film drama entitled “If I Were Jack”.

**Results:**

For each case study, we describe the KT process and strategies that aided development of credible and well-received online content focused on men’s health promotion. In both case studies, the original research generated the inspiration for the interactive online content and the core development strategy was working with a multidisciplinary team to develop this material through arts-based approaches. In both cases also, there is an acknowledgment of the need for gender and culturally sensitive information. Both aimed to engage men by disrupting stereotypes about men, while simultaneously addressing men through authentic voices and faces. Finally, in both case studies we draw attention to the need to think beyond placement of content online to delivery to target audiences from the outset.

**Conclusions:**

The case studies highlight some of the new skills required by academics in the emerging paradigm of translational research and contribute to the nascent literature on KT. Our approach to online KT was to go beyond dissemination and diffusion to actively repackage research knowledge through arts-based approaches (videos and film scripts) as health promotion tools, with optimal appeal, to target male audiences. Our findings highlight the importance of developing a multidisciplinary team to inform the design of content, the importance of adaptation to context, both in terms of the national implementation context and consideration of gender-specific needs, and an integrated implementation and evaluation framework in all KT work.

## Introduction

### Background

The term Knowledge Translation (KT), as conceptualized by the Canadian Institutes of Health and Research (CIHR) in 2000, describes the dynamic interaction between researchers, health care providers, policy makers, and end-users in applying research evidence in practice. While the terminology and definitions of KT vary somewhat across the English-speaking world (eg, [1-3]), the underlying philosophy implied is similar [4]. Its fundamental aim is to move research evidence into action [5] in order to narrow the gap between what is known from research and knowledge syntheses and the implementation of this knowledge by key stakeholders [6].

The field of KT is a young science that is fuelled by the desire of the custodians of public funding to more clearly demonstrate the added value of scientific research for the citizens of society. While this nascent science has much to learn from the more well-established science of intervention design and implementation [7], KT is broader in scope. KT is intended to be the business of all academics, and the activities of KT stretch across the continuum from the development of initiatives for improved and wider dissemination of research beyond academic audiences through to the embedding of new policies and practices in fields such as industrial design, constitutional design, or as in our case, health promotion.

The application of new information communication technologies opens up new mechanisms for knowledge transfer and for breaking down the traditional asymmetry between expert and lay health communication [7,8]. The combined application of information communication technologies to KT is referred to as Technology-Enabled Knowledge Translation (TEKT) [9-11], or more simply e-KT [12]. Ho et al [10] explain how technology can function as a medium for delivery of health research (eg, websites, podcasts, and video conferences) and for evaluation (eg, discussion boards and online surveys). This also includes mobile health, or mHealth, which is the use of mobile tools in distributing health information and accessing health services [13,14]. The shift in the online environment from a passive, unidirectional “read-only” information distribution to a more engaged, multidirectional communication has contributed to its growing popularity (eg, [15-19]). This interactive shift has also led to the emergence of Health Web Science [20], which is not only concerned with how the Web is used for health-related purposes, but also the study of the impact of the Web’s health-related uses on the design, structure, and evolution of the Web itself.

The online environment has particular relevance in the field of men’s health, where historically research has shown that men are less likely to attend primary health care services than women (especially for mental or sexual health problems), yet remain interested in health [21]. While it is acknowledged that men are diverse, researchers have consistently demonstrated that a pervasive aspect of masculinity is a belief that a man’s body and mind should remain strong. This can create cultural barriers to male help-seeking in relation to both mental and physical ill health [22,23]. It is thought that the Internet may have particular appeal for men as a means of help-seeking that does not compromise masculine norms, such as stoicism, and complements their needs for privacy and convenience [24], in large part, because of the private nature and accessibility of electronic mediums [25]. However, the Internet can also be a brokering mechanism to open up help-seeking in relation to health matters [24]. For example, many men use the Internet to access health information in order to maximize the quality of their own care [26-30], and increasingly, both men and women are demanding greater involvement in decisions surrounding their health care [7,19,20].

Despite their promise, however, Internet technologies have not been used to their highest potential for KT on a widespread basis [31-33]. There remains little clear guidance on how to present health-related knowledge online in a way that facilitates understanding among end-users [19,20]. In addition, we know little about how academic researchers, in particular, develop the skills required for successful implementation of online KT strategies or how researchers and stakeholders might work together to ensure maximum impact in this regard. The challenge for many academics faced with such a myriad of new Internet-based technologies is the simple question: What are the strategies I can use to develop the content of my research for lay audiences using Internet-based technologies?

The aim of this paper is to chronicle KT processes used in two distinctly different online case studies in the field of men’s health, delivered in different countries. The case studies we offer share the common ground of aiming to develop content specifically for men’s health promotion and both broadly adopt arts-based approaches to the development of online content using video and film production based on original qualitative research. The primary differences in the case studies is that one (the Men’s Depression: Help Yourself website) is entirely Web-based and the other (If I Were Jack) is not. The latter case study uses an online environment to deliver content into school classrooms. We further understandings of the science of KT by focusing specifically on the design of content for interactive online delivery. Together, the case studies give insight into some of the richness of different methods and approaches while drawing out the broader principles learned. There is currently a dearth of literature that can give other researchers an understanding of some of the choices and challenges behind the online pages that are available to view and a better understanding of how the findings of academic and publicly funded research can be better communicated to the public and used for health promotion.

The structure of the remainder of the paper is as follows. We begin by briefly summarizing some models of KT. We then outline the two case studies, broadly based on one model of KT. We focus on specific choices and decisions reached before drawing conclusions about the benefits and challenges of developing content for interactive online delivery in the field of men’s health—as a means to guiding the future efforts of others. In the discussion section, the processes, practices, and challenges experienced across the case studies are compared and contrasted, illuminating the value of the Internet as a platform for KT in the field of men’s health.

### Models of Knowledge Translation

A number of KT models exist (eg, [5,34]), and most agree that the process should begin with identification of the “gap” between evidence and practice and an analysis of the potential barriers and facilitators of successful KT [32]. Ideally, this evidence should come from quality practice guidelines, systematic reviews, and knowledge syntheses [35] and additionally should engage relevant stakeholders in needs assessment. Indeed, a central underlying feature of KT is the involvement of all important stakeholders (including policy makers, practitioners, and end-users) so that they have shared ownership of the research agenda and KT process [36]. A further key principle following the influence of systems thinking [37,38] is an awareness that the interpretation of information and knowledge is contextually influenced [39,40]. Context-based barriers to KT include issues such as time constraints of end-users, readiness for implementation, and lack of compatibility between the intervention and context.

Graham et al’s integrated KT model [5] is useful in that it presents a “road-map” for those interested in KT from academic research. The process can be summarized into three phases of the knowledge-to-action cycle: (1) Knowledge creation involving background research and knowledge synthesis followed by the design and development of the content, (2) Application of knowledge: adaptation, implementation, and initial evaluations, and (3) Sustainability: How the knowledge can reach the target audience and lead to changes in practice over the medium to longer term. Below we describe how our KT case studies map onto this model before describing some lessons learned through content development.

## Methods

### Case Studies

#### Case Study 1: The “Men’s Depression: Help Yourself” Website

##### Overview

The “Men’s Depression: Help Yourself” (MDHY) website [41] was built with the aim of providing an engaging and interactive online resource focused on men’s depression management. The primary goals were to repackage and share findings drawn from a research program addressing masculinities and men’s depression as a means to (1) support men who experience depression and their families, (2) inform health care providers about how best to identify and treat men’s depression, and (3) raise public awareness and de-stigmatize men’s depression. Given the dramatic increase in the uptake of eHealth resources [42-44], population increases in Canadian-based residents’ daily Internet use (ie, increased from 68 to 80% between 2005 and 2009 [45]), and data indicating that 61% of North American mobile phone owners use smartphones [46], we were excited by the prospects of better understanding how men’s mental health promotion and depression management could be advanced online.

##### Knowledge Creation: Background Research, Knowledge Synthesis, and Content Development

The rates for diagnosed depression are steadily increasing worldwide [47-49], and depression has become a public health concern that is known to significantly increase the risk for suicide [50-52]. In Western countries, men are diagnosed with depression at half the rate of women [53]. Yet, suicide rates for men are up to four times higher than for women [54,55]. Emergent research suggests that the lower reported rates of depression among men may be due to the widespread use of generic diagnostic criteria that are not sensitive to men’s depression [56-58] as well as men’s reluctance to express concerns about their mental health and seek professional health care services [59,60].

This discordant relationship between men’s low rates of depression and high suicide rates prompted our interest to examine the connections between masculinities and men’s depression. Since 2007, we have conducted a series of qualitative research studies with the overarching goal to better understand men’s depression across men of varying ages. Individual interviews with 120 participants (26 college men, 38 middle-aged men and 26 female partners, and 30 older men) revealed an array of experiences linking masculinities and men’s depression, and these findings were chronicled in peer-reviewed journal articles [61-67].

While these publication-based KT efforts constitute dissemination, whereby findings were shared with a broad audience (albeit primarily academics and professionals), we were hopeful that by strategically using interactive Web strategies, we could move toward application [5] through targeting men concerned about depression to raise awareness of our findings and men’s depression more generally. Hence, the MDHY website invites men to “help themselves” as well as reduce stigma and support recovery among men who experience depression.

In an effort to transition our findings toward men-centered interventions, we secured a 1-year end-of-grant KT grant from CIHR (grant# 11R67284). This funded the planning, design, and development of the site, which was launched on May 1, 2013. Based on evidence that interactive Web apps can facilitate engagement online by supporting group interactions and fostering a greater sense of community [68], we incorporated streaming videos, podcasts, online brochures, evites, and a blog to bolster our KT efforts. The site content is accessible from an array of interfaces (eg, personal computers, mobile phones, tablets). Specifically, our overarching aim, to design and develop an interactive KT website, included the development of video clips of 3-4-minutes featuring 10 participants (men who experienced depression, health care providers); journal article author podcasts; interactive plain language booklets to highlight the specific study findings related to college, middle-aged, and older men; e-postcards to invite people to visit the website; and a blog where registered participants could participate by posting comments.

The knowledge synthesis of this background research involved identifying salient experiences related to depression represented in our findings and drawing on principles of men’s health promotion that we had distilled from our ongoing research [67]. We summarized key findings in the brochures and also conducted video interviews with health care providers and individuals experiencing depression to solicit their views about specific articles and findings drawn from our research.

The use of video clips was a purposeful focus to reduce the amount of text and introduce diverse perspectives from both those who experience depression and those who treat men’s depression. We were influenced to include authentic testimonials based on evidence that men respond positively to the sharing of others—especially when they are not themselves under explicit pressure to reciprocate [67]. Bearing this in mind, we captured and edited head and shoulder footage to distil key perspectives about experiencing, as well as treating, men’s depression. All participants were invited to read and respond to some questions related to specific papers detailing findings drawn from the research. Video participants were sourced through researchers’ professional and personal contacts and by contacting participants from earlier men and depression projects (2007-2011) who had agreed to be contacted about future studies. This approach enabled video participants to talk about their perspectives in relation to what others had said and the results of the studies. In this way, opportunities were afforded to participants to differ from, align with, refute, or resonate with what they had read—all as a means to sharing their experiences. This strategy is also known to elicit talk from men who otherwise might be uncertain about the value of such conversations or disclosures [69].

##### Application of Knowledge: Adaptation, Implementation, and Evaluation

Reflecting the substudies in the men’s depression research program, we disaggregated the findings via age groups comprising (1) college men, (2) middle-aged men, and (3) older men as a means of guiding visitors toward the content most relevant to them. Within this context, short videos relating to the men’s experiences and treatment of specific subgroups were bundled and housed under the video tab. Recognizing that a webpage filled with thumbnail images of videos might be off-putting, we also developed e-brochures for each of the age-based subgroups embedding media such as the relevant videos and author podcasts. We also included a “help yourself” button that linked to the videos and our YouTube channel, which also hosted the videos. In offering an array of entry points to view the videos, we hoped to overcome any navigation issues and maximize the exposure of the videos. In terms of evaluation, we were, for the most part, reliant on Google Analytics to draw conclusions about the acceptability of the online content. So, rather than being able to report behavior change or influence exerted by the content, we could describe only the traffic to the site, hypothesizing and comparing the usefulness of the content. In briefly summarizing some of those findings below, we acknowledge the limitations of what can be reasonably claimed.

**Figure 1:**
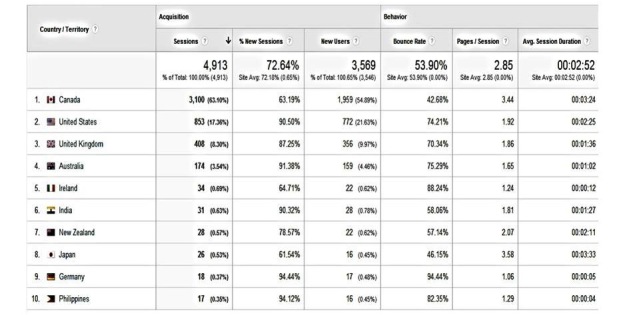
Visitors' pathways by geographic locale.

In the first 12 months (May 2013 through April 2014), there were a total of 4913 visits, resulting in 13,989 page views. Of these visits, 72.64% (3569/4913) were new visits, and the average duration of these visits was 2:52 minutes with a bounce rate (ie, visitors leaving the website directly from the home page) of 53.90%. Direct traffic (ie, visitors typing in the website address because they heard about it) led to a third of visits (32.46%, 1595/4913). Search engine keyword searches accounted for 35.92% of visits (1765/4913) and 31.61% (1553/4913) were referred via other websites (eg, a university men’s health research website [70]) and social media. In terms of the geographical locale (see Figure 1), most of the visits originated from Canada (63.10%, 3100/4913), the United States (17.36%, 853/4913), the United Kingdom (8.30%, 408/4913), and Australia (3.54%, 174/4913)—all English-speaking countries.

The depth of visit as indicated by the page views revealed an average of 2.9 page views per visit. In terms of visitor loyalty, over a quarter of visitors (27.35%, 1344/4913) of visitors returned to the site more than once. This suggests that the site content was somewhat engaging. The most viewed pages were the homepage (31.28%, 4377/13,989), middle-aged men’s page (12.75%, 1784/13,989), followed by the study page (6.60%, 924/13,989). Our blog page was viewed 588 times. Of 33 blog posts, “men’s help seeking for depression—why they do and don’t” generated the most interest; while we received ten comments to our blog posts. In addition, while the evite page was viewed 149 times, only four evites were sent. Perhaps this indicates that visitors used other means to share content through our Facebook or Twitter options, or perhaps the stigmatized representation of depression, as noted earlier, impeded the communication of invitations to others to join in the conversation on the presented materials.

**Figure 2:**
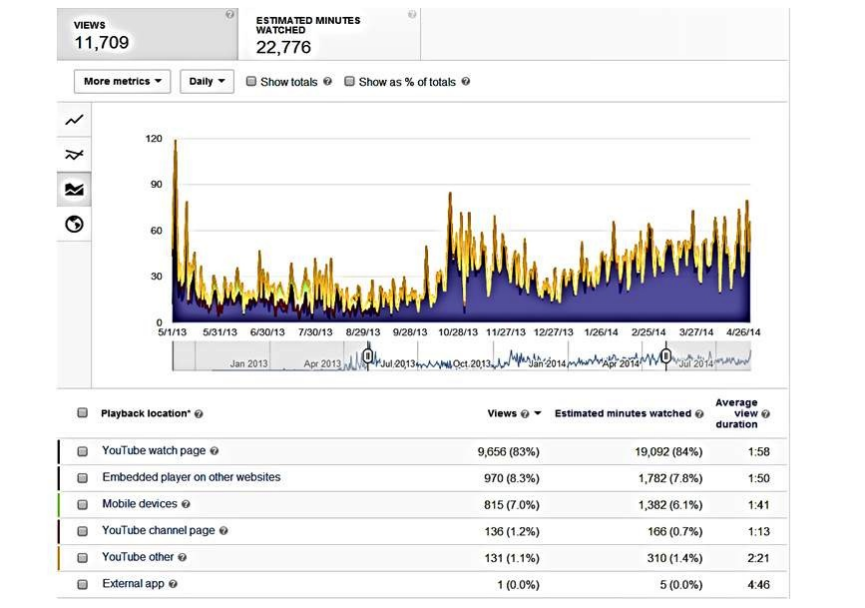
Youtube playback locations.

The 72 videos were viewed 11,709 times in total, and only 970 (8.28%) of these views took place on the MDHY website. The majority (91.71%, 10,739/11,709) took place on our YouTube site/channel (see Figure 2), and 70.74% (7597/11,709) of the YouTube views were made by men 18 years and older (sourced through viewers being logged into their YouTube account at the time of watching). The three most popular videos were the “Men’s depression and recognizing symptoms” (17.47%, 2046/11,709), “Men’s depression and work” (6.17%, 723/11,709), and “Anger and aggression as depressive symptoms” (6.15%, 720/11,709). The podcast page was visited 601 times; yet the 11 podcasts were accessed only 367 times in total. The “Depression, men and masculinities: A review and recommendations” (19.9%, 73/367) and “Masculinities and men’s depression in a northern resource-based Canadian community” (13.6%, 50/367) were the most popular podcasts. In total, the three online brochures were read 1327 times, specifically, College Guys (24.64%, 327/1327), Middle-aged Men (53.96%, 716/1327), and Older Men (21.40%, 284/1327).

In summary, the website did seem to attract men more than women. While all the media platforms used in the MDSY website have the same principle of sharing information and raising awareness of men’s depression, the popularity of our short series of composite videos featuring men’s narratives and reflections was evident, and this finding is consistent with the findings of other research addressing men’s use of the Internet for health promotion [26-30]. That said, some visitors also read the website content more deeply, for example, by accessing the online brochures. This suggests some benefit to providing a range of avenues to access content as a means to broadening the appeal and reach of the website.

##### Sustainability

While sustaining the use of the MDHY website has been supported in part via social media and other outreach strategies to bring men, families, and health care providers to the site, keeping the site fresh with new information and resources is more challenging without recurrent budget. This work, however, laid an important foundation for extending approaches to address men’s depression and suicide [70]. In collaboration with both academic and non-academic partners, a successful Movember grant application has enabled us to extend the online men’s depression help resources.

#### Case Study 2: The Development of an Internet-Based Interactive Video Drama on Teenage Men and Unintended Pregnancy for Implementation Within School Curricula

##### Overview

“If I Were Jack” is a research-informed, culturally sensitive educational resource especially targeting teenage boys to increase their awareness and intentions to avoid an unintended pregnancy. This Internet-based intervention is designed for delivery within sex education or relationship and sexuality education (RSE) in second-level schools to boys as well as girls who are at least 14 years of age. The project website [71] hosts online versions of the resource materials (freely accessible to participating schools) and promotional material including expert videos and practitioner podcasts, which aim to increase the credibility of the resource with users, specifically teachers, young people, and parents. The resource is currently being rolled out in schools in Ireland by the Department of Education, and an Australian version is being used in South Australia by Shine (RSE provider). In Northern Ireland, the acceptability and feasibility of nationwide roll-out is undergoing further investigation through a feasibility cluster randomized controlled trial (trial registration NCT02092480).

##### Knowledge Creation: Background Research, Knowledge Synthesis, and Content Development

The educational resource was developed in response to an identifiable deficit of educational resources that address the sexual health needs of young men [72-77]. Young men are much less likely to receive pregnancy-related RSE, and when they do receive it, they are likely to encounter interventions that are not specifically designed for them [55].

The research knowledge for this KT project began with a systematic review of the literature on adolescent men’s attitudes and decision making in relation to an unintended pregnancy [78]. This was followed by primary research with young men in Ireland examining the psychosocial determinants of their responses to the hypothetical pregnancy scenario [79] and further qualitative research (interviews and focus groups) with educational specialists, teachers, and young people to scope relevant and appropriate RSE materials [80]. The aim of these studies was to redress the longstanding and widespread gender bias in research and interventions on teenage pregnancy, which reports adolescent women’s attitudes and the predictors of their pregnancy resolution choices but largely neglects men’s roles and perspectives [78]. It built upon earlier comparable research in Australia [81-83] described below. However, effective KT also requires knowledge of change mechanisms, therefore, the final stage of the preliminary research also involved learning about developing effective interventions in the field of RSE [84-90] and underlying models of behavioral change [91-94].

The knowledge synthesis of this background research involved the following steps: (1) clearly articulating the need for an educational resource for teenage men, (2) articulating why the school curriculum might be a good place of delivery and thinking through how online delivery would enhance the reach and accessibility of the resource, and (3) developing material based on our background research. While the first case study was able to use interview data to re-develop it for online content, it was the underlying methodology that provided the inspiration for our content. We had already developed an arts-based approach, that is, an interactive film, as a methodology for data collection in the underlying empirical study [79]. Below, we describe how we went on to specifically further develop this methodology as part of an online educational resource.

The design and development phase was partly funded by a KT grant provided by a UK Economic and Social Research Council knowledge exchange research grant (grant #RES-189-25-0300). Central strategies for ensuring successful KT included close consultation with key stakeholders and a focus on optimizing the credibility of the intervention and its acceptability to users including user gate-keepers such as policy makers, health and education experts, and school management, as well as end-users, teachers, pupils, and parents. During the planning phase, we enlisted project partners from the Departments of Health and Education in two countries/jurisdictions (Ireland and Northern Ireland) that would continue to contribute to the KT process throughout and, as we describe further below, enhanced the overall sustainability of the project. Together we decided on the following goal at the outset: to develop an educational resource for post-primary school pupils aged from 14 years addressing teenage men and unintended pregnancy and suitable for delivery as part of the official curricula of schools in Northern Ireland and Ireland.

Together and gradually over time, we also agreed that the components of the resource would be:

- The If I Were Jack interactive video drama (IVD), which asks pupils to put themselves in Jack’s shoes and consider how they would feel and what they would do if they were Jack
- Classroom materials for teachers containing four detailed lesson plans with specific classroom-based and homework activities that include group discussions, role-plays, worksheets, and a parent-pupil exercise
- 60-minute face-to-face training session for teachers wishing to implement the intervention
- 60-minute information and discussion session for parents/guardians delivered by RSE teachers
- Detailed information brochures and factsheets about the intervention and unintended teenage pregnancy in general for schools, teachers, teacher trainers, young people, and parents

In this section, we describe the processes and key choices relating to the first two core components. The production of the Northern Irish and Irish versions of the IVD was based on an earlier Australian version entitled “If I Were Ben” [81]. The Australian research team at Flinders University developed a script for their film by drawing on transcripts of paired and focus group interviews with young men and young women in South Australia on the topic of unintended pregnancy and decision making around keeping the baby and abortion. This original IVD was designed as a research tool for a larger quantitative study [81,82]. While we could have elected to use the Australian version of the IVD, research has suggested the educational advantages of having culturally specific interventions [95] to increase authenticity and to allow for the greatest possibility for young people to identify with the situation. This is also consistent with KT theory, which suggests that knowledge users prefer materials that address their particular realities [33]. Furthermore, our key policy stakeholders, while highly valuing the original Australian IVD, agreed with the merits of a culturally sensitive intervention. We therefore adapted the original script based on consultations with young people in Ireland and Northern Ireland and meetings with our stakeholder partners in the Departments of Health and Education. The main changes arising from the stakeholder meetings were the inclusion of adoption as a third pregnancy resolution option (along with “abortion” and “keep the baby”) and advice on how to deal with particular sensitivities around abortion in both countries. For example, in the section where the young people have to consider all the options, we removed the terms “good things” and “bad things” about abortion, keeping the baby, and adoption in favor of the terms “advantages” and “disadvantages”. The consultation with young people was centered on young people in socially diverse drama groups and drew on the techniques of interactive and embodied drama of Augusto Boal [96] and the Playback Theatre of Jonathan Fox [97]. Using these techniques, young people acted out and creatively adapted the language and scenes to reflect life in Ireland and Northern Ireland for young people, for example, replacing surfing with soccer and driving cars with riding bikes and introduced nuances to friendship relationships. Using university media services, we then shot the movie using Irish actors recruited from youth drama groups and Irish settings. We shot the film “over the shoulder” of the main character to emphasize the possibility of the participants moving through the actor’s world: “You won’t see me but you’ll see the world through my eyes”. Reflecting the importance of culturally sensitive interventions and the historical conflict between Irish and British communities in Northern Ireland, it was necessary to produce Northern Irish and Irish versions using different actors. Excerpts from the IVDs can be viewed on the Jack project website [71].

A further issue that we addressed was the gender-sensitive nature of the intervention. The Australian IVD, when initially developed as a research tool, was for boys only. However, our intervention would be used in mixed-sex classrooms. While we added some questions about the lead female actor: “What might Emma be thinking now?”, we retained the focus on the young man’s perspective in order to use this counterpoint to problematize some of the gender-divisions on the topic and to invite consideration of teenage boys’, as well as teenage girls’, responsibilities.

We then developed the program for the Web using the university’s website content management system to combine videos with basic multiple choice questions. Acting on the advice of project stakeholders and teachers, we disabled the “save” function and background data collection database used in the original research tool. Since the program was being used in schools and due to the sensitive nature of the questions, this was deemed more desirable because it allows young people greater confidentiality. We simultaneously developed paper-based lesson plans to accompany the IVD, which we uploaded to our website. The lesson plans addressed the key learning outcomes of the educational intervention developed through the use of a theory of change logic model and were theoretically informed by the Theory of Planned Behavior [91-93] and the best available evidence regarding RSE practice [84-90]. As these were primarily concerning offline materials uploaded to the Internet, we chronicle the development of these in a separate paper [98].

##### Application of Knowledge: Adaptation, Implementation, and Evaluation

Inspired by our progress, the Australian research team then also converted their IVD for use as an Internet-based intervention in schools in South Australia by developing a partnership with SHINE SA, the primary providers of sex education in schools in South Australia [99]. Establishing partnerships with RSE providers and, in the case of the Irish and Northern contexts, statutory RSE providers, was deemed crucial to mobilize dissemination off the Internet shelf and to provide universal access to the resource to schools. Thus, in Ireland and Northern Ireland, implementation of this resource means opening up two main gates: the first is the statutory custodians of RSE education (and in both countries, this is a mix of both the Departments of Health and Education). The second is the school gate, which we discuss next.

Despite the fact that RSE is a mandatory subject in post-primary schools, implementation of RSE is known to be low priority in some schools [100]. Thus, we also used the project website to post videos of stakeholder testimonies, podcasts with teachers, and information for parents along with stakeholder logos. In particular, it was important for us to reassure schools with a different religious ethos that although all pregnancy resolution options are discussed, none are presented as optimal and that the resource reflected the legal situation in relation to the availability of abortion in each country. The intervention also allowed schools to express their school ethos in relation to abortion within the context of the overall discussion materials. Finally, in terms of implementation, we sought approval from the custodians of the schools’ Internet server in each country to allow schools to access the materials—otherwise the child safety blocks might prevent access. In summary, implementation of this Internet-based resource relied upon it appearing “safe”, “sensitive”, and “sanctioned”.

While the resource will undergo further evaluation in terms of its effectiveness in increasing young people’s understanding of the issues as well as increasing their intentions to avoid an unintended pregnancy using randomized controlled trial (RCT) methodology, data from a mixed-methods cross-sectional study confirms that the educational intervention is already achieving key educational and health promotion outcomes. Table 1 presents results based on a sample of 746 boys drawn from a stratified random sample of schools in South Australia and Ireland. These results suggest that nearly three-quarters of the Ireland sample (n=284) and Australian sample (n=271) achieved increased awareness in relation to the issue of teenage pregnancy, and nearly three-quarters in both country samples (n=284 in Ireland; n=266 in Australia) said it helped them to think they should avoid an unintended teenage pregnancy. The key aims of the planned (RCT) evaluation will be to test the feasibility of the intervention for use in different UK contexts and to adapt it as necessary. If the intervention is found to be both acceptable and effective, it has the potential to benefit all pupils aged 14-16 in Northern Ireland and the rest of the United Kingdom.

**Table 1:** Knowledge-users’ evaluation of the impact of the educational resource [83].

Impact on adolescents	Ireland (n=360) Strongly agree/agree, %	Australia (n=386) Strongly agree/agree, %
Got me involved in Jack’s/(Ben’s) situation	72	60
Made me think about issues I hadn’t thought about before	79	70
Helped me understand the effect an unplanned pregnancy would have on a guy like me	85	72
Made me think that I should never get myself in that situation	79	69
Made me aware that I could talk to a counseling service if I were in Jack’s situation	84	n/a

##### Sustainability

Throughout this paper we have referred to collaboration with stakeholders, which we would argue was key, along with having a very sound evidence base, to achieving the degree of sustainability that has been achieved (so far, roll-out through a state provider in Ireland into the curriculum, roll-out through a private provider of RSE in Australia, and further testing of the resource in Northern Ireland). However, it is worth opening up the black box of *how* we collaborated for other researchers interested in KT. While we described underlying research with young people and timed consultations with young people in developing this resource, for us the central plank of collaboration was with the statutory stakeholders of RSE. An application to a third body for a KT grant was our reason for talking with key personnel within the Departments of Health and Education in Ireland and Northern Ireland, asking them to come on board and work with us to develop an educational resource suitable for delivery in the curriculum. The “coming on board” also happened incrementally, as one stakeholder recommended another and, in some cases, recruited others. Once the project started, meetings with stakeholders happened face to face twice a year in the university. Once stakeholders were involved, we then fostered a team approach to the development of the resource, making any changes the stakeholders suggested because we recognized them as the experts in implementation. The diverse range of stakeholders from two government departments and two different countries around the table meant that issues raised were debated in the context of conversations between experts rather than as ultimatums for the researchers. In summary, the lessons we learned in terms of recruiting and collaborating with stakeholders are as follows. Collaborating with researchers to produce evidence-based practice resources is a fruitful approach with policy makers. Researchers need to be aware that policy makers, especially statutory policy makers, usually operate in more highly governed workplaces than academics do and authorization for their involvement takes time and effort. Building in travel money for stakeholders may be essential. Face-to-face meetings build trust and, finally, having the stakeholders and not the researchers represent the joint work, for example in online videos, is a signifier of knowledge translation.

## Results

### Creating Engaging Interactive Materials

In both case studies, the original research generated the inspiration for the interactive online content. While the limitations of qualitative research in changing health practices is often acknowledged [101], a key strength of qualitative research is the ways that it can generate language, imagery, and intonation to meaningfully communicate key health messages to others. In a scoping review of arts-based health research, Boydell et al [102] highlighted that the arts and qualitative research share common ground, recognizing the significance of rich description and the subjective nature of human experience. Bringing these arts-based approaches to online KT to fruition involved a multidisciplinary team of researchers together with video, media, Internet, and drama experts but remained a research-led enterprise. For the MDHY website, content and characters challenged the stigma men can experience talking about depression and/or help-seeking. Videos were made of men who experienced depression and their family, as well as health care providers who work with men with depression. In these videos, participants reflected on their own experiences as well as their reactions to the overall findings of the Men and Depression studies, thereby re-telling their stories and sharing their advice to help men to help themselves. These stories became the basis of the online interaction, provoking almost 5000 visits in the first year of the site, the majority of which were by men, to listen in on the conversation.

In relation to the If I Were Jack website, we built on the earlier qualitative research conducted by a different team of researchers that had led to the development of a computer-based interactive video drama on a young man’s experience of an adolescent pregnancy for use in further primary research. Through the use of interactive theater techniques in youth drama groups, we adapted the script of the original IVD, reproduced two further versions and adapted them for online delivery within classrooms. These arts-based approaches to translating qualitative research into online health promotion content drew on psychological research that suggests the value of episodic thinking—an exercise of the imagination that allows one to “pre-experience” an event in order to adaptively prepare [103,104] (eg, from Jack: “I want you to imagine you’re me”), as well as research that suggests the value of understanding our own self-care through an understanding of the experiences of others [105,106].

### Developing Gender and Culturally Sensitive Interventions

Both research teams set out to engage men by disrupting stereotypes about men’s stoicism and reluctance around sharing vulnerabilities with others. We attempted to engage with men less through humor or sexual imagery and more through producing authentic voices and faces that our target audiences could identify with. Recognizing the important role women can play in men’s health [107], and in acknowledgment of findings from our own research of the value in breaking down gender stereotypes of men’s health [66,79], we also wanted to engage women in the websites, so in both cases, there is clear representation of women and invitations for women to participate. For example, the interactive video on the If I Were Jack website is designed to be used by females as well as males and invites females to also imagine being a male in this situation. Both males and females are asked to further consider what Jack’s girlfriend, Emma, would also be thinking.

The issue of designing culturally sensitive content was also something both teams thoughtfully considered. For the *Jack* team, we decided it was necessary to make two different versions of Jack (an Irish version and a Northern Irish version, in addition to the original Australian version) because identification with the lead character is central to the exercise of the imagination: “You’ll see the world through my eyes, you’ll be me. I want you to make some decisions for me because things are about to get tough around here”. The MDHY website had the opportunity to include more faces and more stories, and the team sought to represent the diversity of Canadian society. The team also designed flexible and user-friendly routes through the website to stories and related resources designed for specific target groups of men, such as older men and college men.

### Achieving Sustainability of Knowledge Translation Over the Longer Term

Both case studies drew attention to the necessity to think beyond placement of content online to delivery to targeted audiences from the outset. The lesson here is the need to work collaboratively with stakeholders, gate-keepers, and potential partners. For the *Jack* team, the issue was ensuring the website could get beyond the school gate and this required careful collaboration in content development with the main custodians of sex education/RSE in schools in our target countries. In the case of the MDHY website, it meant developing a community of practice with other major charities and provincial providers of health promotion so as to achieve an online and offline reference community. While we were clearly thinking of implementation from the outset of the KT process, and both case studies have achieved footholds in online and offline communities, arguably now researchers should be thinking about KT and implementation processes from the outset of research process.

## Discussion

### Summary

We have described how our KT case studies map onto the model of the knowledge-to-action cycle and its three phases. Some lessons learned through content development have also been discussed.

To date, much of the published research on KT has focused on developing a unified definition of KT [1,3] or developing general models and road-maps to guide researchers [5,108], leaving unanswered questions about how to creatively develop online KT content from research. This paper adds flesh to the bones of this science and illustrates how basic social science research can be transitioned into accessible, interactive, informative, and user-friendly online content to support KT. In this paper, we have demonstrated how we used a model of KT to inform a pathway for development of e-KT, while illustrating some of the challenges we encountered including choices to be made in making appealing content that was culturally and gender-sensitive, and in achieving sustainability using two case studies that span a number of countries. The work also builds on men’s health research and especially emerging research on how best to deliver eHealth to men [12,109,110].

### Limitations

If we were to start all over again, we would refer to Normalization Process Theory, which is a nascent science building on implementation theory, which, in its application to eHealth, seeks to explain and predict the success or failure of the implementation and integration of new eHealth technologies into everyday practices [7]. While this theory is targeted to researchers developing and implementing interventions rather than the broader activities encompassed under KT, as noted earlier, the field of KT has much to learn from this scholarship in relation to the processes of translating research into practice. More generally, a limitation of developing online content for KT is the necessity to keep content fresh and relevant and to regularly monitor the target audience to see if it is attuned to their needs. Again, this implies partnerships with non-academic audiences to sustain knowledge translation. Finally, in this paper we have not presented a longitudinal or comparative systematic evaluation of these online men’s health KT strategies. The focus has been on the design of the content rather than on rigorous evaluation. Although we have presented evaluation data that can suggest the impact of the Internet-based men’s health interventions, future papers will more fully develop this aspect of the research [111].

### Conclusions

There has been limited success with conventional approaches in engaging men in health promotion. Our case studies illuminate (1) the importance of working with a multidisciplinary team of academics, creative practitioners, stakeholders, and the target group itself to inform the transition of research findings into meaningful and accessible online content to improve men’s health, (2) the power of qualitative research with men in leading the direction of creatively developing gender and culturally sensitive communication with men about health issues, (3) the importance of engaging stakeholders from the outset to secure relevant adaptation to context and delivery to targeted audience, and (4) the importance of thinking about KT strategies from the outset of a research project and developing an integrated process and impact evaluation framework in all KT work.

## Abbreviations

CIHR: Canadian Institutes of Health and Research
IVD: interactive video drama
KT: knowledge translation
MDHY: Men’s Depression: Help Yourself website
RSE: relationship and sexuality education
TEKT (or e-KT): technology-enabled knowledge translation
